# Towards 5G: A Photonic Based Millimeter Wave Signal Generation for Applying in 5G Access Fronthaul

**DOI:** 10.1038/srep19891

**Published:** 2016-01-27

**Authors:** S. E. Alavi, M. R. K. Soltanian, I. S. Amiri, M. Khalily, A. S. M. Supa’at, H. Ahmad

**Affiliations:** 1 Faculty of Electrical Engineering, Universiti Teknologi Malaysia, 81310 UTM Johor Bahru Malaysia; 2 Photonics Research Centre, University of Malaya, 50603 Kuala Lumpur, Malaysia; 3 Institute for Communication Systems (ICS), home of the 5G Innovation Centre, Department of Electronic Engineering, University of Surrey, Guildford GU2 7XH, U.K

## Abstract

5G communications require a multi Gb/s data transmission in its small cells. For this purpose millimeter wave (mm-wave) RF signals are the best solutions to be utilized for high speed data transmission. Generation of these high frequency RF signals is challenging in electrical domain therefore photonic generation of these signals is more studied. In this work, a photonic based simple and robust method for generating millimeter waves applicable in 5G access fronthaul is presented. Besides generating of the mm-wave signal in the 60 GHz frequency band the radio over fiber (RoF) system for transmission of orthogonal frequency division multiplexing (OFDM) with 5 GHz bandwidth is presented. For the purpose of wireless transmission for 5G application the required antenna is designed and developed. The total system performance in one small cell was studied and the error vector magnitude (EVM) of the system was evaluated.

Due to fast revolution in mobile technology, wireless technology has been evolving from 1G to 4G. Seamless integration of cellular networks such as GSM and 3G, WLAN and Bluetooth was the main focus of 4G systems^1^ and now 4G technology is also reaching maturity. Currently researchers are working on defining next generation of wireless communication, i.e., 5G. Emphasizing on small cell concepts, network speed and capacity enhancement and introducing new communications technologies are among the most important announcements about future 5G^2^. Improving the speed and capacity is required to make the communication for potentially billions of wireless devices feasible. Wireless access network (WAN) at millimeter wave bands (30–300 GHz) has large bandwidth which offers an alternative for high speed indoor/hotspot communication to be utilized for 5G. To support the combination of small cells and mm-wave radio for future 5G access, fiber-optic communication plays an important role in both the backhaul and fronthaul networks. According to^3^ optical access network (OAN) is required to be scalable to support the expected 5G deployment goals by 2020: 1–10 Gb/s at the user terminal; 100 Gb/s for the backhaul truck; 1 Tb/s for metro transport and 1 Pb/s for the core transport. Here, the view of the next-generation 5G which is the integration of high speed WAN-OAN is illustrated in Fig. 1.

As can be seen from Fig. 1, to make an end-to-end transport network all different types of WAN are connected to the fiber optic. In one hand, OAN provide very-high-speed Internet access for fiber to the x (FTTX) applications through optical line terminal (OLT). On the other hand, high-speed and convenient mm-wave and small cell WAN is utilized. For the high speed WAN many bands including the local multipoint distribution service at 28–30 GHz, the license-free band at 60 GHz, and the E-band at 71–76 GHz, 81–86 GHz, and 92–95 GHz are become available for 5G^4^,^5^,^6^.

For generating the high-frequency mm-wave electrical signals, using conventional electronics becomes less financially attractive, therefore, there is a high concern to directly generate mm-wave signals in the optical domain. As a consequence in this work, the photonic generation, modulation and distribution of 60 GHz frequency band signals to be applied in 5G have been suggested and demonstrated. Essentially, a photonics based mm-wave is a laser beam consisting of two or more coherent longitudinal modes with frequency spacing equal to the wanted mm-wave. As the longitudinal modes beat with each other in the photodiode, the required electrical mm-wave could be generated.

The generation of microwave signals from optical sources has long been a substantial area of interest due to its large potential for a variety of applications in communications^7^,^8^,^9^,^10^,^11^.

A wide variety of techniques have been demonstrated for optically mm-wave generation, e.g., based on dual-mode laser^12^, dual lasers with different wavelengths locked by optical phase locking and/or injection phase locking^13^,^14^, mode locked lasers^15^, on optical external modulation (EM)^16^,^17^,^18^,^19^,^20^,^21^,^22^ optical heterodyning techniques^23^,^24^ or on approaches based on nonlinear properties including four-wave mixing (FWM)^25^,^26^,^27^, and stimulated Brillouin scattering^28^,^29^. There is also some methods based on an optical frequency combs (OFC) that is generated by i.e. gain switching a distributed feedback laser (DFB)^30^. This comb generation technique offers flexibility as the free spectral range (FSR) of the optical combs possess tunability within a 20 GHz range and provides excellent signal to noise ratio.

Since the two longitudinal modes generated by a dual-mode laser or two separate lasers have low coherency, the mm-wave generated by their beating has poor spectral purity^31^,^32^. While mode locked lasers can generate a wide bandwidth frequency comb, they suffer from cavity complexity and lack tunability of the FSR due to a fixed cavity length. Moreover, the optical linewidth of the individual comb lines may be relatively large and the imperfect phase coherence between two optical tones can cause significant phase noise on the generated mm-wave signal^33^. The longitudinal modes generated by the nonlinear effects have higher coherency, but higher pump powers are required and their conversion efficiencies are low^34^. Moreover, since the Brillouin frequency shift is fixed in the fiber, the generated optical mm-wave is frequency limited^35^. In case of EM, modulators have large insertion losses particularly when cascaded. Coupled with the modulation efficiency and the instability induced by bias drift, the use of the EM technique can be complex^36^.

Dual wavelength fiber laser (DWFL) is one of the interesting approach to generate radio frequency (RF) signals at mm-wave frequencies^37^,^38^ and terahertz sources^39^,^40^. Erbium doped fiber (EDF) is the most commonly used gain medium in fiber laser setup in regions of 1.5 micron including multi-wavelength laser. However, strong mode competition induced by the homogeneous broadening^41^ has become the main challenge to achieve stable multi-wavelength oscillation in room temperature. Achieving dual wavelength generation in the 1.5 μm wavelength region with EDFL is challenging due to strong mode competition caused by homogeneous gain broadening in the gain media. Various approaches for overcoming this setback, such as FWM^42^, polarization hole burning^43^ and cascaded stimulated Brillouin scattering^44^, have been reported for Thulium-doped fiber laser (TDFL), Ytterbium-doped fiber (YDF) and EDF. Various methods have been proposed to realize the multi-wavelength operation at room temperature by reducing cross-gain saturation and suppressing mode competition. These include polarization hole burning effect^45^, frequency-shifted feedback^46^, cascaded stimulated Brillouin scattering^47^, four wave mixing^48^, etc. Recently, the authors have demonstrated a tunable spacing dual wavelength using a photonic crystal fiber (PCF) as a Mach-Zehnder interferometer utilizing a polarization dependent loss (PDL) effect^49^. This DWFL shows high power stability with a very narrow linewidth. The use of PCF brings advantages of flexibility and wavelength-dependent characteristics, which make the material almost an ideal choice as a wavelength selective filter for EDF^50^. The work that is described in this paper had the central principle of generating stable DWFL with specific inter-spacings in order to obtain CW 60 GHz radiation of high quality and stability.

For the purpose of wireless transmission of the generated mm-wave signal in 60 GHz spectrum it is required to utilize the antenna suitable for 5G applications. The authors in^51^ proposed a new four-element dense dielectric patch array antenna at 28 GHz for future 5G short-range wireless communications with relatively large size, complex structure, good impedance bandwidth and 16 dBi gain using EBG and dielectric superstrate. In this work an appropriate antenna to be utilized in 60 GHz is designed to be utilized in our proposed 5G system. Moreover, in this report, the generated DWFL provides comparative outputs using only a very short-length of PCF (only 10 cm long). The proposed generated DWFL not only utilizes a very simple and compact ring laser cavity, but also generates coherent and tuneable DWFL. Consequently, the RF signal generated by the proposed setup is tuneable, simple, and more stable compared to the other discussed methods. This has not been reported before, and the information would be of substantial benefit to the scientific and engineering community in the sense that it would allow for the development of a compact and robust dual-wavelength source that can be easily and safely used to generate RF radiation in real-world settings.

## Experimental Setup For Generating 60 Ghz Rf Signal

**Figure 2:**
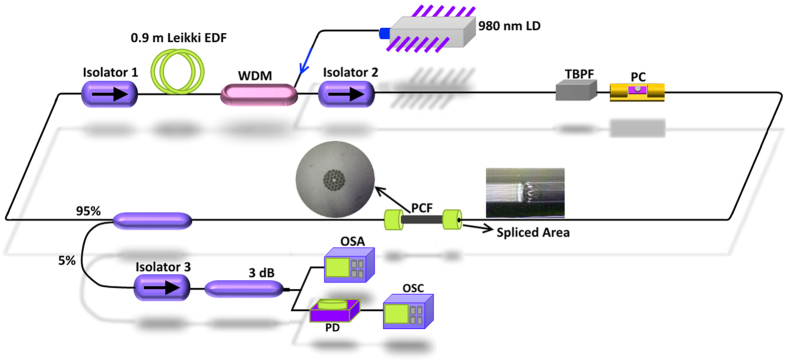
Experimental setup to generate stable dual-wavelength fiber laser.

The configuration of the DWFL system in 1.5 micron region is schematically shown in Fig. 2. The DWFL consists of a 980 nm pump laser diode (LD), a 980/1550 nm wavelength division multiplexing (WDM), a 0.9 m Likkie EDF, a tunable band-pass filter (TBPF) with 1 nm bandwidth, isolators, a polarization controller (PC), a very short length of 10 cm PCF, and a 5:95 fiber based coupler.

The LD is directed into the fiber laser ring cavity through a WDM to provide excitation to the gain medium. TBPF is used to limit or confine the oscillation of dual wavelength laser in a narrow spacing. The isolators are used to assure unidirectional operation of the laser as to achieve a more stable lasing condition. The PC is inevitably exploited to adjust the polarization of light propagating inside the ring cavity. PCF plays the main role in the setup to stabilize the DWFL. The micrograph of the PCF cross-section structure is shown in the inset in Fig. 2. The PCF surrounded by air holes with 5.06 μm diameter and separation of 5.52 μm between holes. The coupler is used to direct part of the laser power out of the laser cavity for measurement, analysis and application.

The output end of the PCF was connected to the input port of coupler, with the 95% output port then connected to isolator 1. This isolator was further linked to the Leikki EDF gain medium that possessed an NA of 0.21 to 0.24, mode field diameter of 5.7–6.6 at 1550 nm, and absorption coefficient of 84 dB/m at 980 nm. The other end of the EDF was subsequently connected to a WDM fused coupler. Thereafter the 980 nm port was coupled to a LD operating at approximately 80 mW. The signal port of the WDM was connected to another isolator, labeled as isolator 2 which is attached to TBPF in series with a PC. The TBPF acted as a window to tune the generated dual wavelength output and had specifications of 0.8 nm narrow bandwidth, 0.05 nm high tuning resolution, a broad tuning range of 1535–1565 nm, and a low insertion loss typically around 1.5 dB. Laser output was continuously monitored by means of an optical spectrum analyzer (OSA) connected to a 3 dB coupler that was linked, by way of an isolator labeled isolator 3, to a fiber coupler on the main optical ring. This main circuit coupler was responsible for diverting 5% of the laser output towards isolator 3. Another branch of the 3 dB coupler with one port linked to the OSA was then connected to an oscilloscope (OSC) through a photodiode to show RF spectrum generated by beating of the DWFL.

**Figure 3:**
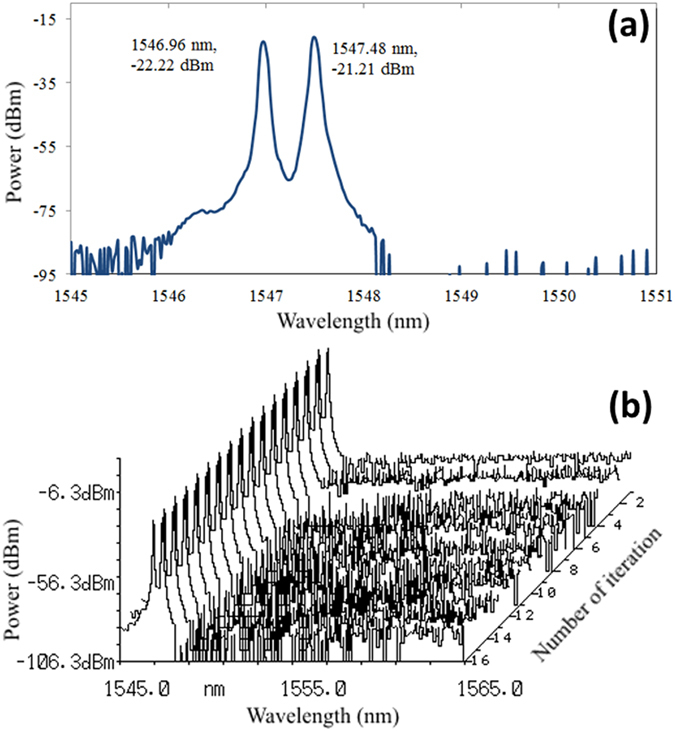
(**a**) Optical spectrum of DWFL lasing at wavelengths 1546.96 nm and 1547.48 nm and (**b**) the stability of achieved DWFL over 160 minutes with the interval scan of every 10 minutes.

Figure 3(a) shows the experimental result of the DWFL with 0.52 nm spacing between two wavelengths lasing at wavelengths 1546.96 nm and 1547.48 nm. The stability of the achieved DWFL over 160 minutes is shown in Fig. 3(b) with the interval scan of 10 minutes. The displacement of the output lasing can be achieved by fine tuning of the PC due to the polarization dependence of the laser inside the ring cavity. The adjustment of the PC will rotates the polarization states and allows continuous adjustment of the birefringence within the ring cavity to balance the gain and loss of the lasing wavelengths. By achieving dual wavelengths laser with similar peak powers, a stable DWFL can be obtained. The PC is then fine-tuned to obtained dual wavelength laser with almost similar peak powers at −22.22 dBm and −21.21 dBm at wavelengths 1546.96 nm and 1547.48 nm respectively as shown in Fig. 3.

## Results For 60 Ghz Signal Generation

The unique properties of PCF, such as wide range single mode operation, dispersion flexibility and large mode area, have been investigated recently^52^, and the inherent flexibility in particular puts PCF in contention for use as an interferometer. Interferometers employing PCF also can be wavelength-selective filters^50^ due to the wavelength-dependent characteristics afforded by PCF. A constructed compact interferometer using a 10 cm length of few-mode PCF spliced on each end to SMF, as shown for one side in the image labeled “spliced area” in Fig. 2. Manually splicing the PCF and SMF fiber at two points in series led to an inevitable collapse of the PCF air holes and creation of a very simple Mach-Zehnder interferometer. The first collapsed region served to diffract the traversing fundamental mode, and consequently allow core and cladding modes to become excited within the few modes PCF section. A portion of the fundamental core mode can be coupled to a single or several cladding modes in the PCF. Phase shifting within a physical length, L, of the PCF is a product of dissimilar effective refractive indices of core and cladding modes. As the effective refractive index of the cladding is smaller than that of the core, separate optical paths that correspond to arms of the Mach-Zehnder interferometer can be achieved. The fundamental and cladding modes accumulate a phase difference along the PCF due to different phase velocities, and this phase difference depends on the length of PCF and the wavelength of the guided light. Cladding modes recouple to the core mode upon reaching the second collapsed area of the PCF. Since the phase difference and the phase velocities are wavelength dependent, the optical power transmitted by the interferometer will be minimum at certain wavelengths and maximum at the others. The separation between consecutive peaks of a two-mode interferometer is given by 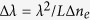, where λ represents the source wavelength, L is the length of the PCF between the two splices, and Δ*n*_*e*_ symbolizes the effective refractive indices difference between the core and cladding modes. The optical paths established via the core and the cladding modes behavior within the PCF play the role of arms of a Mach–Zehnder interferometer, and the collapsed points perform as couplers that split or combine light in the interferometer arms.

**Figure 4:**
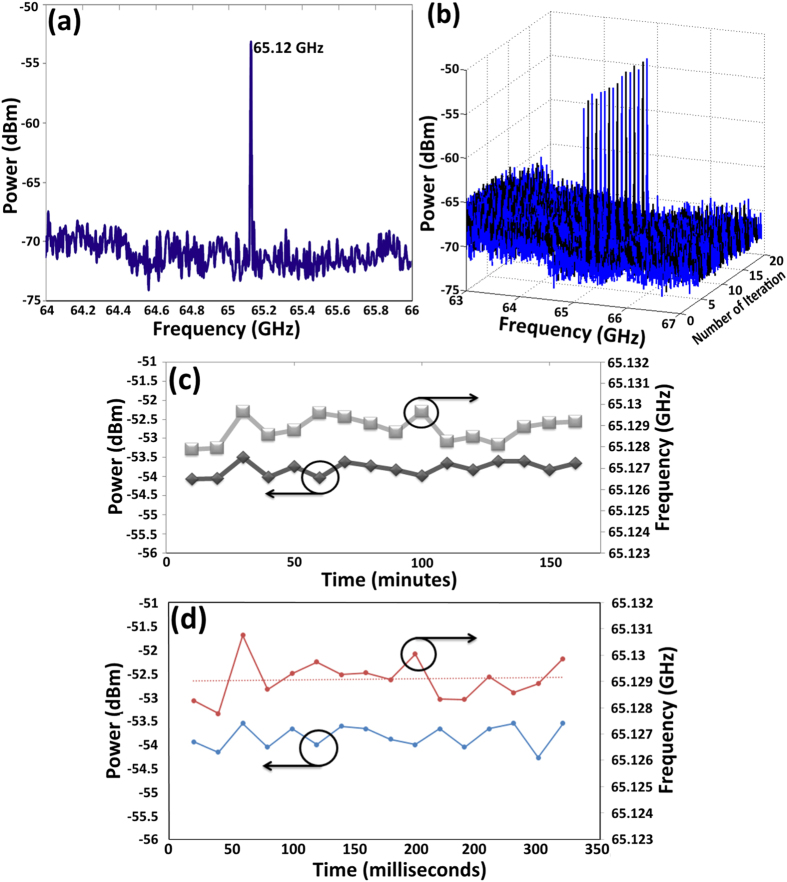
(**a**) The beating frequency of 65.12 GHz in the RFSA, (**b**) the stability of generated RF over 160 minutes with the scan interval of 10 minutes and (**c**) power and frequency fluctuation of the generated RF as a function of time recorded in 160 minutes and (**d**) 320 ms in the scan interval of every 20 ms.

The microwave generation is subsequently determined by connecting the dual-wavelength output to the photo detector and taking measurements with the RFSA. The RF spectrum is shown in Fig. 4(a) as having a frequency of 65.12 GHz. As described earlier, the 60 GHz frequency band has the potential to support very high data rate wireless communication applications. Considering the limited data rates offered by current wireless LAN (802.11a, 11b, etc) and mobile networking systems, it is anticipated that future higher data rate wireless LAN and mobile networking technology could occur in the 60 GHz band. Furthermore, in the UK these millimetre wave bands are expected to relieve congestion and reduce demand for spectrum in frequency bands below 20 GHz^53^ that would further attract commercial interest. The frequency drift associated with these transceiver devices is an important factor in specifying frequency band requirements. According to several test reports on 60 GHz equipment available on the Federal Communications Commission’s website, the worst case frequency deviation was recorded to be 57 MHz from the nominal frequency when the transmitter was operated between −20 °C to 50 °C. Noting the above, there is a need to avoid interference being caused to adjacent frequency bands due to frequency drift of equipment operating in the 59.3–64 GHz bands. It is therefore proposed to introduce 100 MHz guard bands to protect adjacent band services from excessive drift in operating milimiter wave transceiver (MWT) equipment. The frequency tolerance of a crystal or oscillator is defined as the initial deviation of the crystal or oscillator frequency as compared to the absolute at 25 °C. The frequency stability over temperature is defined as the frequency deviation compared to the measured frequency at 25 °C over the defined operating temperature range (i.e. 0 °C to +70 °C). For instance in 60-GHz radio regulation in Japan, allowable frequency deviation is limited by +/−500 ppm which is corresponding to frequency deviation of +/−30 MHz.

Therefore, here, the signal stability is investigated by scanning the RF spectrum for every ten minutes. The RF power and frequency stabilities are recorded as shown in Fig. 4(c). It is found that the frequency drifts between 65.1265 GHz and 65.1274 GHz with 0.00077% maximum deviation from the average. Figure 4(d) provides the RF power and frequency stabilities during 320 ms in the scan interval of every 20 ms. The carrier frequency can be further stabilized by placing the fiber spool in a temperature controller or using polarization maintaining fiber (PMF). RF power stability is important in the implementation of oscillators and carriers. The measured average peak power is at −52.55 dBm and its peak power fluctuation is ±0.49 dB. Fluctuations in the millimeter wave power can only be reduced with the enhancement of laser stability. The measured power is low due to the bandwidth limitation of the photodetector.

## 60 Ghz Antenna Design

**Figure 5:**
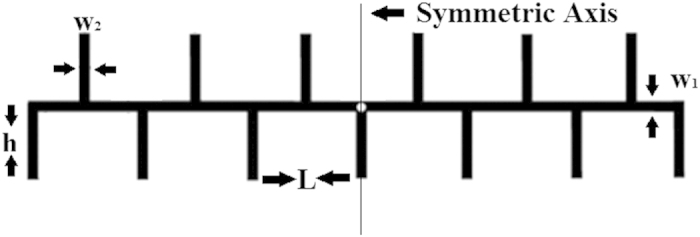
Schematic of the proposed antenna.

Microstrip Comb Array Antenna (MCAA) has been designed to operate at 60 GHz for 5G application. The MCAA shows broader impedance bandwidth and lower cross-polarized radiation as compared with the conventional microstrip grid array antennas. The MCAA is designed not as a travelling-wave, but a standing-wave antenna. As a result, the match load and the reflection-cancelling structure can be avoided, which is important, especially in the millimeter-wave frequencies. The structure of designed antenna is presented in Fig. 5. In this work, the Ansoft-HFSS software is used for different analysis and parametric simulations. In all designs, a single-layer Rogers RT/duroid 5880 substrate (εr = 2.2, h = 0.787 mm & tanδ = 0.001) is used. Dimensions are optimized by using parallel parametric studies and tabulated in Table 1.

**Figure 6:**
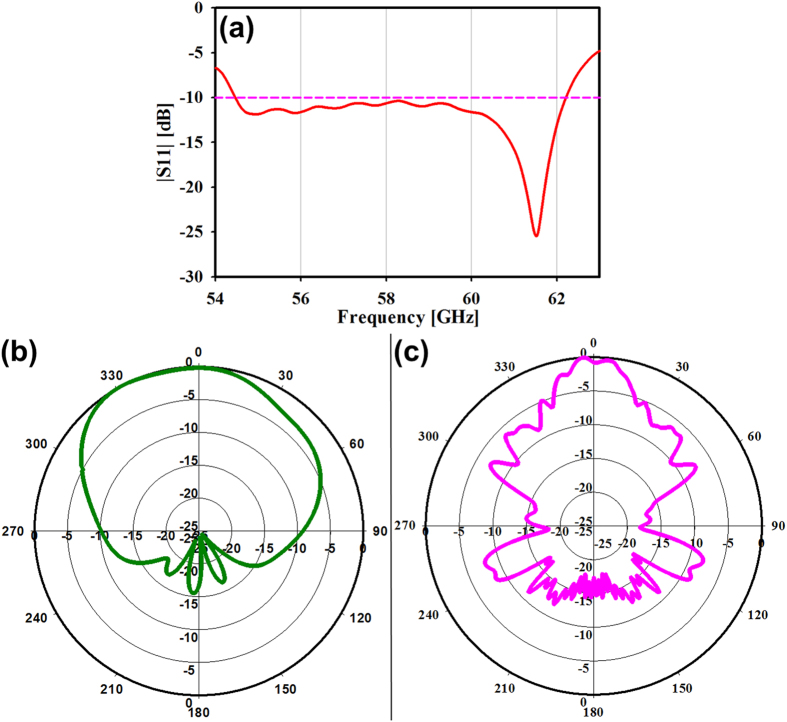
(**a**) Measured |S_11_| of the proposed antenna for 5G application (**b**) E-plane & (**c**) H-plane radiation patterns of the proposed antenna at 60 GHz.

Proposed antenna has a gain of 23.6 dB at 60 GHz which is greater than 5G antenna’s gain requirement^54^. In this design not only high gain with an acceptable bandwidth around 7.8 GHz has been obtained but also the side loop level is below −20 dB. Measured |S_11_| versus frequency is shown in Fig. 6(a). As can be seen clearly from this figure the proposed MCAA achieves −10 dB impedance bandwidth of 7.8 GHz from 54.43 to 62.23 GHz and proves that the bandwidth is more than enough bandwidth has been covered by the proposed antenna for 5G application as 1 GHz bandwidth is enough for 5G application^55^.

Figure 6(b,c) show the co-polar radiation patterns of the proposed antenna in the *H* and *E* planes at 60 GHz, respectively. It must be noted that a narrow beam in the *H-* plane with the 3-dB beamwidth of 20° and a broad beam in the *E-* plane with 3-dB beamwidth of 90° has been observed.

## System Setup

**Figure 7:**
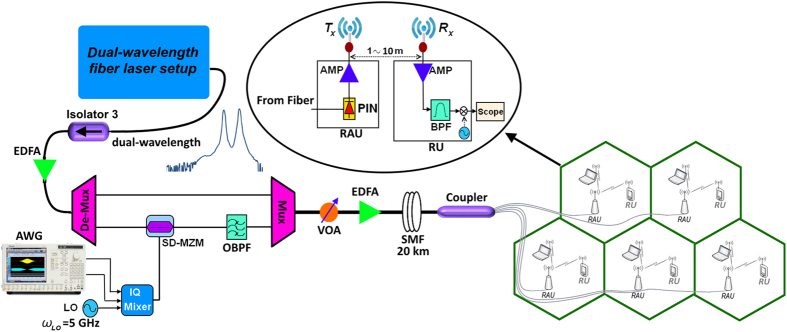
System setup.

Figure 7 shows the system design. First, using the proposed DWFL system discussed in Section 2 the dual wavelength signal with 65.12 GHz frequency distance between each wavelength is generated.

Then in order to utilize the generated carriers in 5G application we used the OFDM signal to be transmitted in a 5G small cell. For this purpose the OFDM signal is generated using arbitrary waveform generator (AWG) and it was up-converted to IF region using the I/Q mixer with local oscillator of f_LO_ = 5 GHz. The OFDM signal parameters are listed in Table 2.

**Figure 8:**
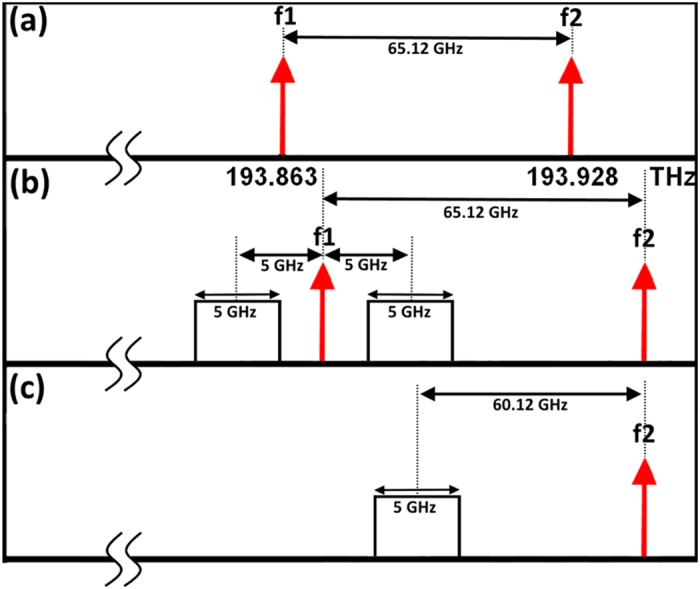
Wireless RF signal generation for 5G.

As shown in Fig. 8, the lower carrier from DWFL system was modulated by the OFDM signal using double side band techniques with an single-electrode Mach–Zehnder modulator (SD-MZM) while the upper carrier is kept unmodulated. The optical filter with a centre frequency of 193.86 THz and 15 GHz bandwidth is used to remove the lower carrier and the lower side band of the OFDM (LSB-OFDM); therefore, only the upper side band of the OFDM (USB-OFDM) is multiplexed by the unmodulated upper carrier they are 60.12 GHz apart from each other.

Next, the multiplexed signal is amplified by an erbium-doped fibre amplifier (EDFA) and transmitted over an SMF link. In the remote antenna unit (RAU), the modulated OFDM signal and unmodulated carrier that are 60.12 GHz apart are beaten to photodetector; therefore, after photodetection, the 60.12-GHz mm-wave OFDM signal is amplified using a low noise amplifier (LNA) and then transmitted to the 5G antenna with 23-dBi gain designed in previous section. Increasing the number of channels in optical communication systems will eventually result in the usage of optical signal demultiplexing components with greater values of optical attenuation. Additionally to this, when transmitted over long distances, the optical signal is highly attenuated, and therefore, to restore the optical power budget it is necessary to implement optical signal amplification. At the choice of signal amplification method for the wavelength division multiplexing (WDM) systems the preference is given to the class of (EDFAs). Therefore, due to the low power of the generated DWFL, the use of the EDFAs is necessary in order to accommodate the power budget. These amplifiers are low-noise, almost insensitive to polarization of the signal and can be relatively simply realized^56^. Besides providing gain at 1550 nm, in the low-loss window of a silica fiber such amplifier allows achieving such gain in wider RF band. To ensure the required level of amplification over the frequency band used for transmission it is highly important to choose the optimal configuration of the EDFAs, as the flatness and the level of the obtained amplification, and the amount of EDFA produced noise are highly dependent on each of the many parameters of the amplifier. Moreover, to achieve a stable RF frequency resulted from beating of the generated DWFL, EDFA decreases the power fluctuation of DWFL and provides more stable RF signal.

After wireless transmission, the OFDM signal is received by a second 5G antenna with 23 dBi gain. Subsequently, at the receiver unit (RU), the received signal is amplified using LNA (57–64 GHz), and then, electrical down-conversion is performed in the 60 GHz-band. The down-converted signal is further amplified in the frequency range of 0.7–12 GHz and then captured by Oscilloscope operated at a sampling rate of 50–100 GS/s. Baseband digital signal processing including one-tap equalization, demodulation, demapping, and error vector magnitude (EVM) computation are performed offline. The EVM is defined as follows:


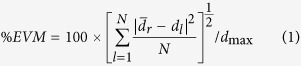


where 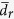 and 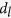 are the received and ideal symbols, respectively, and 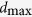 is the maximum symbol vector in the constellation. Here, N is the number of 16-QAM symbols^55^.

## System Performance

**Figure 9:**
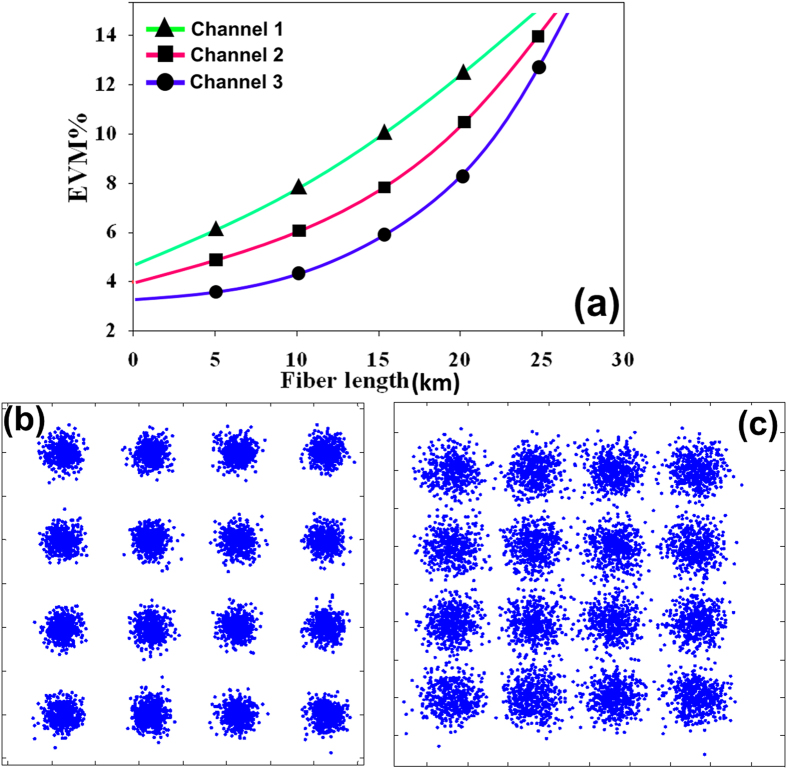
EVM performance and constellation diagram related to different optical link lengths and 6 m wireless link.

First, the system EVM performance was tested based on the variation of the optical link length for a fixed wireless distance (5 m). Second, the EVM of the system based on the variation of the wireless link distance between 1–10 m over a 20-km SMF was calculated. Figure 9 shows the EVM performances of 16-QAM OFDM signals for different lengths of the SMF transmission link and different wireless channels. The optical power is fixed at 0 dBm using variable optical attenuator (VOA). The constellations for 10 km and 20 km for wireless channel (2) also shows a successful transmission for 5 m wireless link at the threshold EVM of 10%^55^.

**Figure 10:**
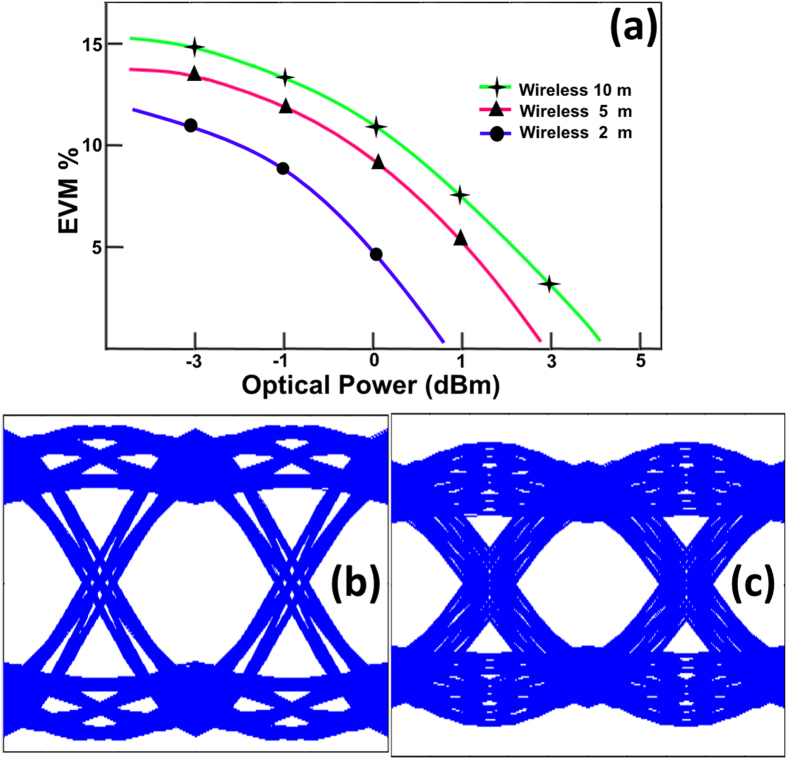
(**a**) EVM performance for different wireless link distances, (**b**) eye diagrams for 2 meters wireless distance, (**c**) eye diagrams for 5 meters wireless distance.

In the second case shown in Fig. 10, the dependence of system performance on the wireless distance was investigated. Here, for fiber length of 20 km the received optical power was varied from −3 to 5 dBm, and for three different wireless distances (10, 5 and 2 meters) the EVM was calculated. With an increase in the wireless distance, the free-space path loss causes degradation of the EVM performance. It can be seen that the system can support a maximum wireless transmission distance of 10 m for the power penalty of 0.7 dBm. The eye diagrams also show the successful transmission for two wireless distances of 2 and 5 m.

## Conclusions

In this study, a DWFL system has been demonstrated through detailed discussions of the mm-wave signal generation system for 5G applications. Data transmissions are achieved using a 5 GHz bandwidth OFDM signal centred on 60 GHz. For the purpose of wireless transmission in 5G small cell network a 60 GHz antenna was designed and fabricated. The efficiency of the system was evaluated using EVM measurement curve. Based on the results it can be concluded that the system with a 16-QAM OFDM signal can be received by the end user with acceptable EVM over a 20 km optical fibre and a 10-m wireless link with an optical power penalty of 0.7 dBm.

## Additional Information

**How to cite this article**: Alavi, S. E. *et al.* Towards 5G: A Photonic Based Millimeter Wave Signal Generation for Applying in 5G Access Fronthaul. *Sci. Rep.* **6**, 19891; doi: 10.1038/srep19891 (2016).

## Figures and Tables

**Figure 1:**
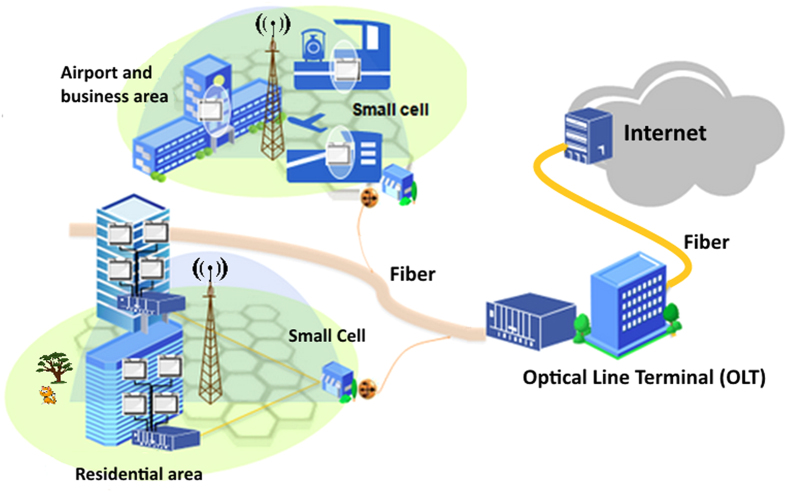
Next-generation converged optical-wireless access networks in 5G (drawn by authors, SE Alavi and IS Amiri).

**Table 1:** Optimized antenna dimensions for the proposed antenna.

Parameter	L	w_1_	w_2_	h
Value	3.01 mm	0.4 mm	0.6 mm	1.505 mm

**Table 2:** OFDM signal parameters.

AWG	Parameters
Tektronix AWG7122C	f_s_ = 12 GHz
	FFT_size = 512,
	Enabled carriers = 426
	OFDM_BW_ = 5 GHz
	CP Length = 1/16 frame
