# Author Correction: Towards 5G: A Photonic Based Millimeter Wave Signal Generation for Applying in 5G Access Fronthaul

**DOI:** 10.1038/s41598-020-69377-1

**Published:** 2020-07-22

**Authors:** S. E. Alavi, M. R. K. Soltanian, I. S. Amiri, M. Khalily, A. S. M. Supa’at, H. Ahmad

**Affiliations:** 1 Faculty of Electrical Engineering, Universiti Teknologi Malaysia, 81310 UTM Johor Bahru, Malaysia; 2 Photonics Research Centre, University of Malaya, 50603 Kuala Lumpur, Malaysia; 3 Institute for Communication Systems (ICS), home of the 5G Innovation Centre, Department of Electronic Engineering, University of Surrey, Guildford, GU2 7XH U.K.

Correction to: *Scientific Reports* 10.1038/srep19891, published online 27 January 2016

The text of the Article does not make it clear that the RF results reported in Figure 4 are based on the simulation of the generated DWFL. The DWFL was generated experimentally and the corresponding RF beating was simulated.

As a result, in the section ‘Results For 60 Ghz Signal Generation’,

“The microwave generation is subsequently determined by connecting the dual-wavelength output to the photo detector and taking measurements with the RFSA. The RF spectrum is shown in Fig. 4(a) as having a frequency of 65.12 GHz."

should read:

"The microwave generation could be subsequently determined by connecting the dual-wavelength output to the photodetector and taking measurements with the RFSA. The simulation results for the corresponding RF spectrum is shown in Fig. 4(a) as having a frequency of 65.12 GHz."

Additionally, in the same section,

“Therefore, here, the signal stability is investigated by scanning the RF spectrum for every ten minutes. The RF power and frequency stabilities are recorded as shown in Fig. 4(c).”

should read:

"Therefore, here, the signal stability is investigated by scanning the simulated RF spectrum for every ten minutes. The RF power and frequency stabilities are shown in Fig. 4(c) are simulated based on the stability of the generated DWFL."

Additionally, in the same section,

"The measured average peak power is at − 52.55 dBm and its peak power fluctuation is ± 0.49 dB."

should read:

"The average peak power is at − 52.55 dBm and its peak power fluctuation is ± 0.49 dB."

Furthermore, in the same section,

“The measured power is low due to the bandwidth limitation of the photodetector.”

should read:

"In general, RF power measurement has low efficiency due to the bandwidth limitation of the photodetector."

Finally, the legend of Fig. 4,

“(a) The beating frequency of 65.12 GHz in the RFSA, (b) the stability of generated RF over 160 min with the scan interval of 10 min and (c) power and frequency fluctuation of the generated RF as a function of time recorded in 160 min and (d) 320 ms in the scan interval of every 20 ms.”

should read:

"(a) The simulated beating frequency of generated DWFL at 65.12 GHz, (b) the stability of simulated RF over 160 min with the scan interval of 10 min based on the stability of the generated DWFL and (c) power and frequency fluctuation of the generated RF as a function of time in 160 min and (d) 320 ms in the scan interval of every 20 ms."

